# Identification of H7 as a novel peroxiredoxin I inhibitor to induce differentiation of leukemia cells

**DOI:** 10.18632/oncotarget.6763

**Published:** 2015-12-26

**Authors:** Wei Wei, Chunmin Ma, Yang Cao, Li Yang, Zhimin Huang, Dongjun Qin, Yingyi Chen, Chuanxu Liu, Li Xia, Tongdan Wang, Hu Lei, Yun Yu, Min Huang, Yin Tong, Hanzhang Xu, Fenghou Gao, Jian Zhang, Ying-Li Wu

**Affiliations:** ^1^ Hongqiao International Institute of Medicine, Shanghai Tongren Hospital/Faculty of Basic Medicine, Chemical Biology Division of Shanghai Universities E-Institutes, Key Laboratory of Cell Differentiation and Apoptosis of The Chinese Ministry of Education, Shanghai Jiao Tong University School of Medicine, Shanghai 200025, China; ^2^ Department of Hematology, Xinhua Hospital Shanghai Jiao-Tong University School of Medicine, Shanghai, 200092, China; ^3^ Institute of Oncology, Shanghai 9th People's Hospital, Shanghai Jiao Tong University School of Medicine, Shanghai 200011, China; ^4^ Department of Hematology, Shanghai First People's Hospital, Shanghai Jiao-Tong University School of Medicine, Shanghai, 200081, China

**Keywords:** peroxiredoxin, leukemia, cell differentiation, reactive oxygen species

## Abstract

Identifying novel targets to enhance leukemia-cell differentiation is an urgent requirment. We have recently proposed that inhibiting the antioxidant enzyme peroxiredoxin I (Prdx I) may induce leukemia-cell differentiation. However, this concept remains to be confirmed. In this work, we identified H7 as a novel Prdx I inhibitor through virtual screening, *in vitro* activity assay, and surface plasmon resonance assay. Cellular thermal shift assay showed that H7 directly bound to Prdx I but not to Prdxs II–V in cells. H7 treatment also increased reactive oxygen species (ROS) level and cell differentiation in leukemia cells, as reflected by the upregulation of the cell surface differentiation marker CD11b/CD14 and the morphological maturation of cells. The differentiation-induction effect of H7 was further observed in some non-acute promyelocytic leukemia (APL) and primary leukemia cells apart from APL NB4 cells. Moreover, the ROS scavenger N-acetyl cysteine significantly reversed the H7-induced cell differentiation. We demonstrated as well that H7-induced cell differentiation was associated with the activation of the ROS-Erk1/2-C/EBPβ axis. Finally, we showed H7 treatment induced cell differentiation in an APL mouse model. All of these data confirmed that Prdx I was novel target for inducing leukemia-cell differentiation and that H7 was a novel lead compound for optimizing Prdx I inhibition.

## INTRODUCTION

In acute myeloid leukemia (AML), immature cells are blocked abnormally at an early stage of their development and fail to differentiate into functional mature cells. The introduction of all trans-retinoic acid into the acute promyelocytic leukemia (APL) therapy validated the concept of differentiation therapy [1]. However, differentiation therapy remains limited to APL treatment. Despite the discovery of many differentiation agents, such as vitamin D and 1, 25-dihydroxy vitamin D3 [2–4], differentiation therapy failed in most non-APL leukemia cases. Therefore, a deep understanding of the molecular mechanisms of leukemia cell differentiation, as well as identifying novel differentiation agents, is urgently required.

In the mammalian hematopoietic system, the reactive oxygen species (ROS) levels are apparently important in controlling myeloid precursor differentiation. Accumulating data have shown that ROS-modulating agents, such as arsenic trioxide, DFO, and tetrandrine, may regulate leukemia differentiation by increasing ROS levels [5–10]. Therefore, identification of small molecules exhibiting ROS-modulating activity may provide new tool in investigating the underlying mechanisms of leukemia-cell differentiation and in developing new therapy for leukemia.

Peroxiredoxins (Prdxs) are a superfamily of small non-selenoperoxidases that contain essential catalytic cysteine residues and use thioredoxin as an electron donor in reducing hydrogen peroxide (H_2_O_2_) [11]. At least six mammalian isoforms, including two-cysteine (Prdxs I–IV), atypical two-cysteine (Prdx V), and one-cysteine (Prdx VI) Prdxs have been identified [12–13]. Prdx I is abundant in cells, constituting a total of 0.2%–1% of soluble protein in cultured mammalian cells [14]. Emerging data have shown that Prdx I levels are elevated in several human cancer cells and tissues, such as oral, esophageal, pancreatic, follicular thyroid, breast, and lung cancers. The elevated Prdx I proteins enhance the aggressive survival phenotype of cancer cells and confer an increased resistance to chemotherapy and radiotherapy [15–19]. We recently demonstrated that adenanthin, a natural diterpenoid, could induce differentiation of leukemia cells through the Prdx I/ROS axis [20–21]. We then proposed that Prdx I represents a novel target in developing differentiation agents. However, this concept need to be further confirmed.

Using virtual screening, along with *in vitro* and cellular assay, we identified in this study that H7 is a novel Prdx I inhibitor. We further demonstrated that H7 induces leukemia-cell differentiation *in vitro* and *in vivo*. We validate the notion that Prdx I is a novel target that induces leukemia-cell differentiation and H7 is a novel lead compound for developing Prdx I inhibitors.

## RESULTS

### H7 is a novel Prdx I inhibitor

**Figure 1:**
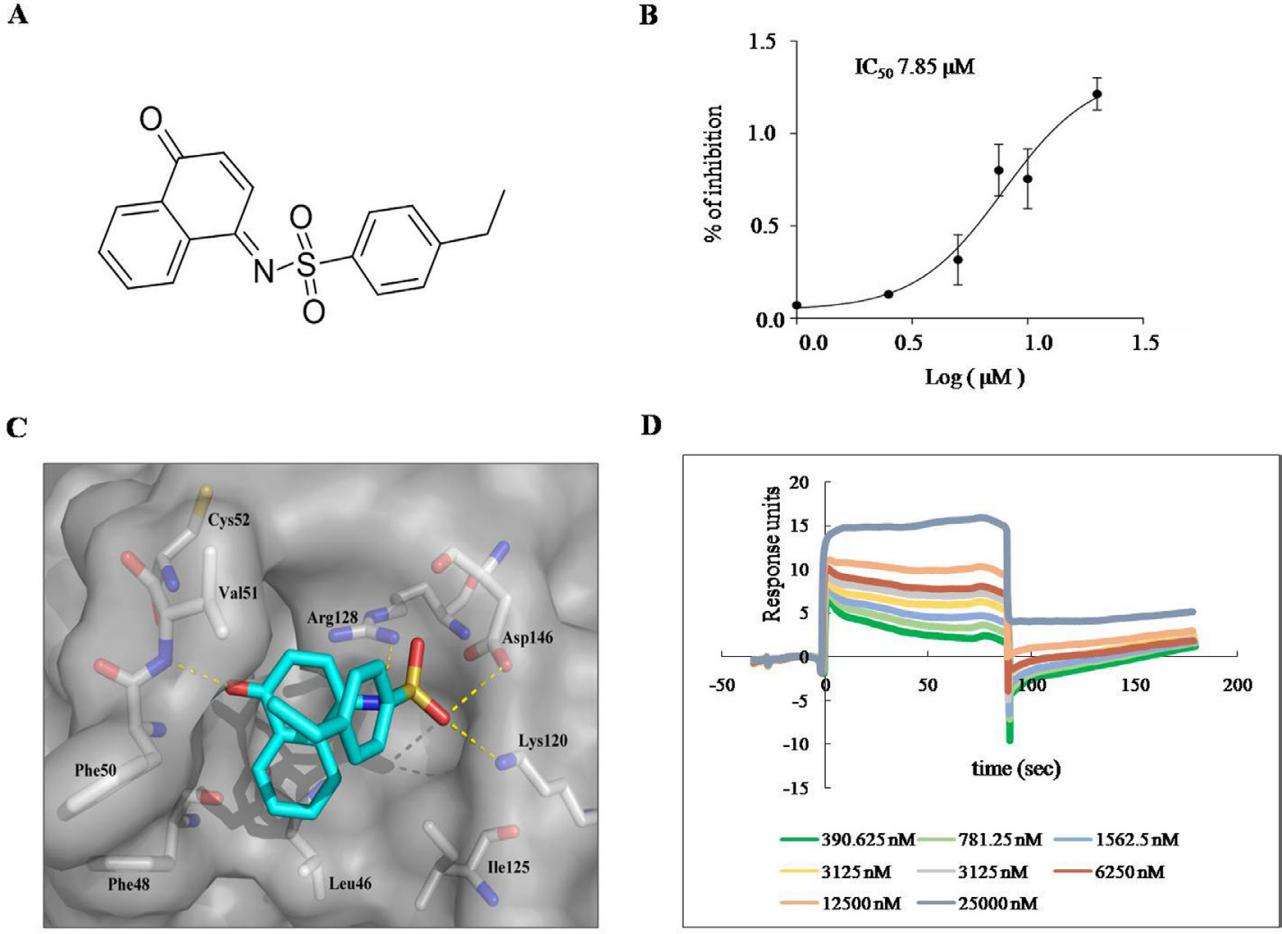
H7 inhibits Prdx I catalytic activity **(A)** Chemical structure of H7. **(B)** The recombinant Prdx I protein was incubated with the indicated concentration of H7 for 1 h, and its peroxidase activity was monitored for 1200 s. The IC_50_ of H7 on Prdx I was calculated by the Graphpad prism software. All values represent the means ± S.D. of three independent experiments. **(C)** Binding model of H7 and Prdx I. The molecular surface of Prdx I protein is shown in gray and the H7 molecule is shown as sticks with light blue carbons. **(D)** SPR analysis of the binding between Prdx I and H7. The recombinant Prdx I protein was immobilized on an activated CM5 chip. H7 was then flowed across the chip at increasing concentrations. The chip was regenerated between concentrations using 2 M glycine. Dose-dependent binding of H7 was observed across the concentration range.

We used virtual screening, along with *in vitro* Prdx I activity assay, to identify the novel Prdx I inhibitors. In the virtual screening, the candidate compounds from different scaffolds were selected and their potency for Prdx I inhibition was analyzed using the *in vitro* Prdx I activity assay. Among the compounds, H7 (Figure 1A) showed the most potent inhibition of Prdx I activity and was thus selected for further investigation. The IC_50_ of H7 on Prdx I activity was 7.85 μM (Figure 1B). Moreover, docking study showed that H7 is buried in a pocket composed of Leu46, Phe48, Phe50, Val51, Cys52, Lys120, Ile125, Arg128, and Asp146. Moreover, The sulfonyl and carbonyl group of H7 form four hydrogen bonds, of which make it to stably interact with and inhibit Prdx I, with both side chains of Lys120, Arg128, Asp146 and main chain of Val51, respectively (Figure 1C). These data suggest that H7 is a novel Prdx I inhibitor.

The binding between H7 and Prdx I was further evaluated by surface plasmon resonance (SPR) assay using a biacore platform. The sensorgrams showed that H7 rapidly associated and disassociated from the immobilized Prdx I at a dissociation constant of 1.57 μM (Figure 1D). Moreover, the response signal during the dissociation phase returned to the baseline level for H7, indicating complete dissociation of the compound from Prdx I. These data suggest that H7 is non-covalently bound to Prdx I.

### H7 interacts with Prdx I in cells

**Figure 2:**
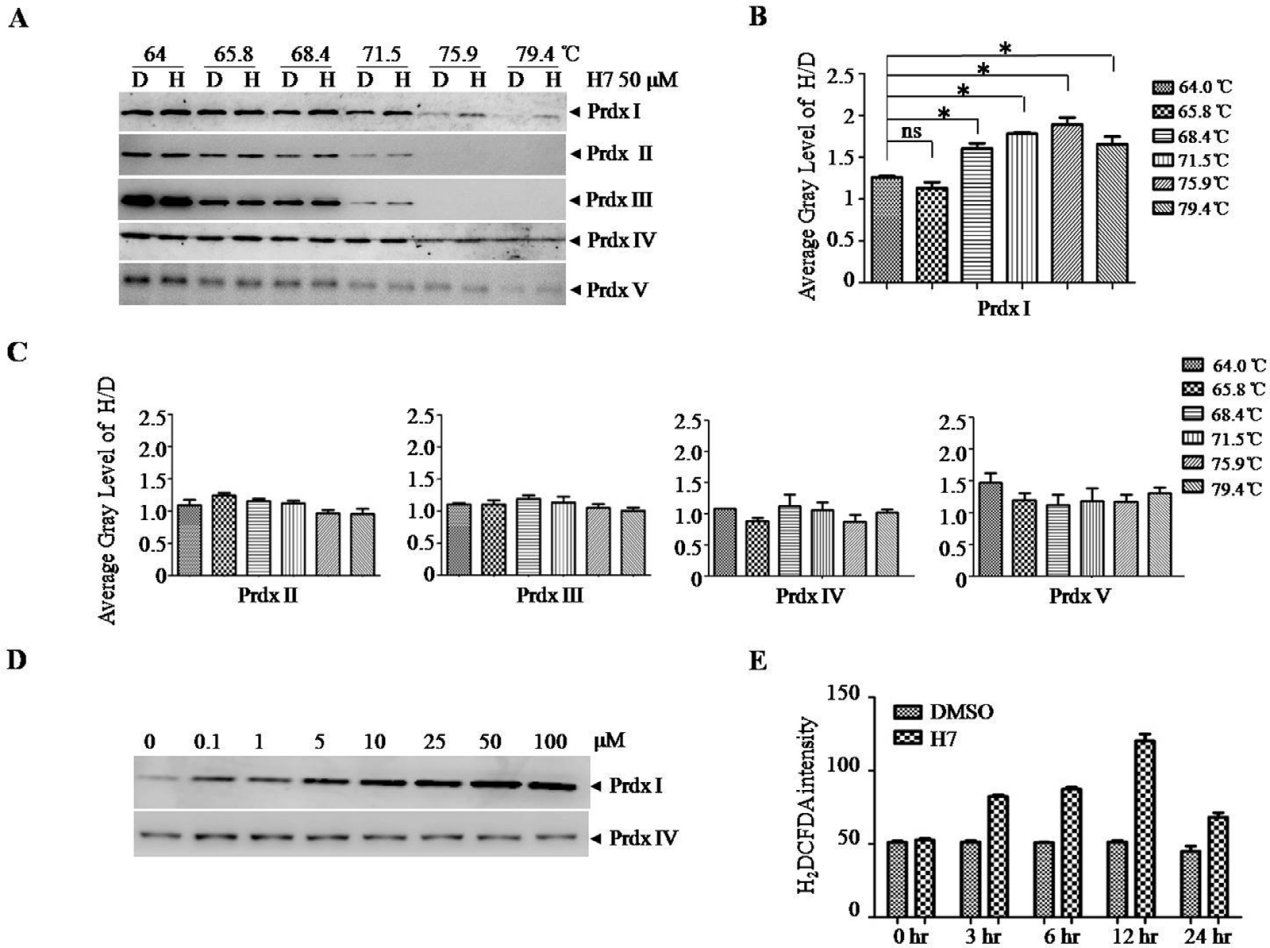
H7 interacts with Prdx1 in cells **(A)** The NB4 cell lysate was incubated with DMSO or H7 at different temperatures for 3 min followed by centrifugation at 20 000 g. The supernatant was subjected to Western blot analysis of the Prdxs I–V proteins. **(B–C)** The intensity of the bands of the Prdxs I–V proteins were quantified by the Quantity One software, and the relative ratio of H7- and DMSO-treated bands (H / D) was displayed. All values represent the means ± S.D. of three independent experiments. *p < 0.05. Prdx IV was used as a loading control. **(D)** The NB4 cell lysate was incubated with DMSO or different H7 concentrations at 75.9°C for 3 min followed by centrifugation at 20,000 g. The supernatant was subjected to Western blot analysis for the Prdx I protein. Prdx IV was used as a loading control. **(E)** The NB4 cells were treated with H7 for different times, and the intracellular ROS were evaluated by DCFDA staining. Each experiment was repeated at least three times.

To investigate whether the interaction between H7 and Prdx I observed *in vitro* does occur in cells, we performed cellular thermal shift assay (CETSA). CETSA is a newly developed method of measuring the direct binding of protein with its ligand in cells; this technique is based on the concept that the direct binding of a small molecule to its target protein may increase the stability of proteins in response to heat [22]. Figure 2A and 2B showed that the addition of H7 but not DMSO into the cell lysates increased the stability of Prdx I at different temperatures. Prdx I was highly stabilized at 75.9°C. However, H7 did not significantly affect the stability of Prdxs II–V, indicating the relative selectivity of H7 on Prdx I (Figure 2C). Moreover, the stabilization effect of H7 on Prdx I is dose dependent (Figure 2D). Given that Prdx I functions as a H_2_O_2_ scavenger, we subsequently determined whether H7 treatment increases ROS level in NB4 cells. The ROS level in the H7-treated NB4 cells gradually increased, peaked after 12 h, and then declined after 24 h (Figure 4E). These data demonstrate that H7 could reach its target protein Prdx I in a biologically relevant setting, leading to increased ROS level.

### H7 induces NB4 cell differentiation

**Figure 3:**
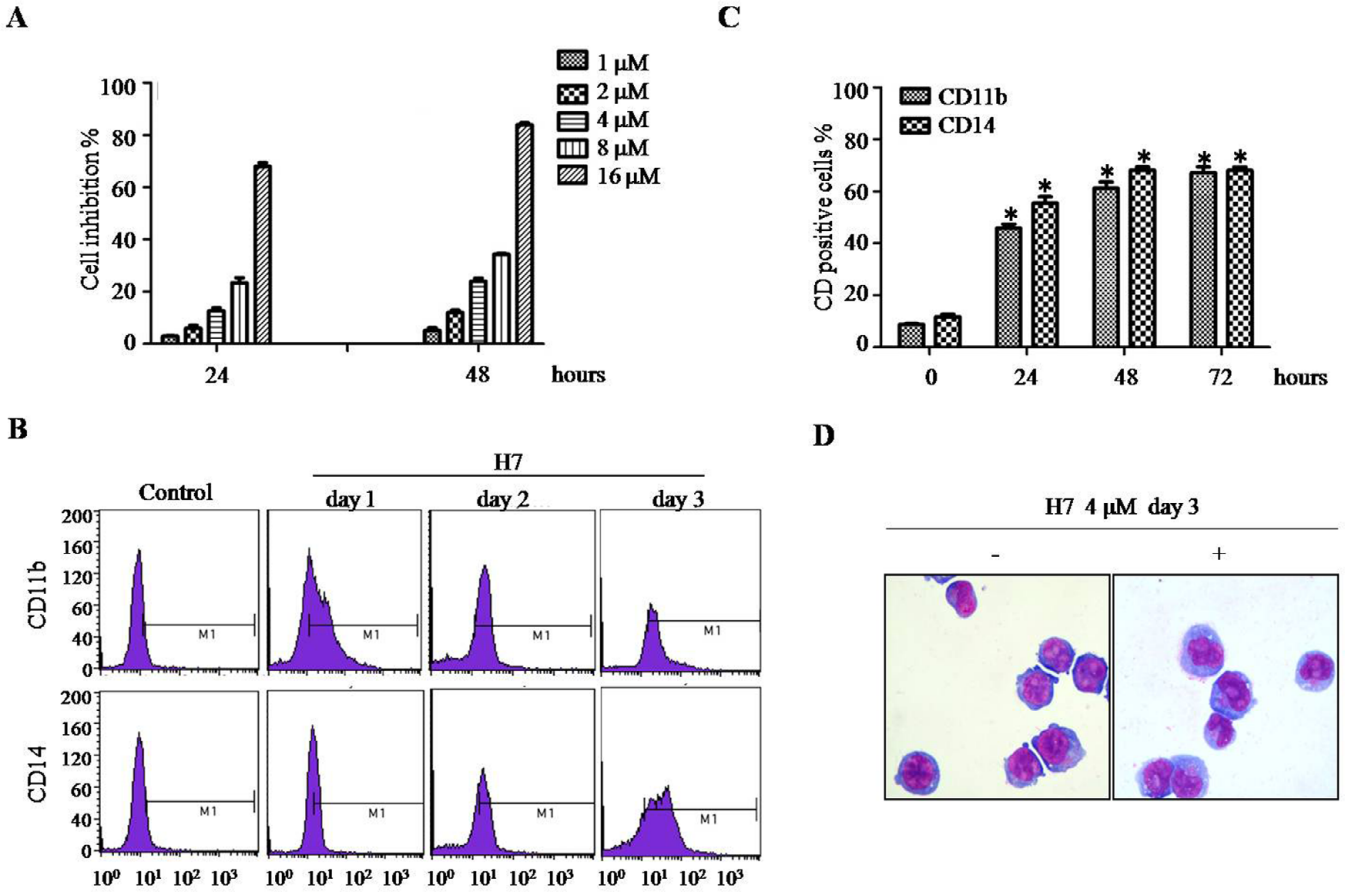
H7 induces partial differentiation of NB4 cells **(A)** The NB4 cells were treated with different H7 concentrations for 24 and 48 h. Cell proliferation was determined by trypan blue exclusion assay. **(B–C)** The NB4 cells were treated with H7 for 1, 2, and 3 days, and the CD11b and CD14 expression levels were determined by FACS. The representative histogram and percentages of CD11b and CD14 are shown in (B) and (C), respectively. All values represent the means ± S.D. of three independent experiments. *p < 0.05 compared with the control group. **(D)** The NB4 cells were treated with H7 for 3 days, and cell morphology was examined by Wright's staining and light microscopy (100 ×). Each experiment was repeated at least three times.

Given that targeting Prdx I by adenanthin induces leukemia-cell differentiation [20], we then determined whether H7 can also induce leukemia-cell differentiation. H7 exerts dose- and time-dependent growth inhibition effect on NB4 cells (Figure 3A). At 4 μM, H7 significantly inhibited NB4 cell growth without obvious loss of cell viability. Thus, we selected this H7 concentration in subsequent experiments. The NB4 cells were treated with H7 for 24, 48, and 72 h, and then cell differentiation was monitored. H7 treatment increased the percentages of CD11b- and CD14-positive cells (Figure 3B, 3C); morphologically, H7 also reduced the nuclei/cytoplasm ratio, indicating monocyte differentiation of NB4 cells (Figure 3D).

### Prdx I is essential in H7-induced leukemia-cell differentiation

**Figure 4:**
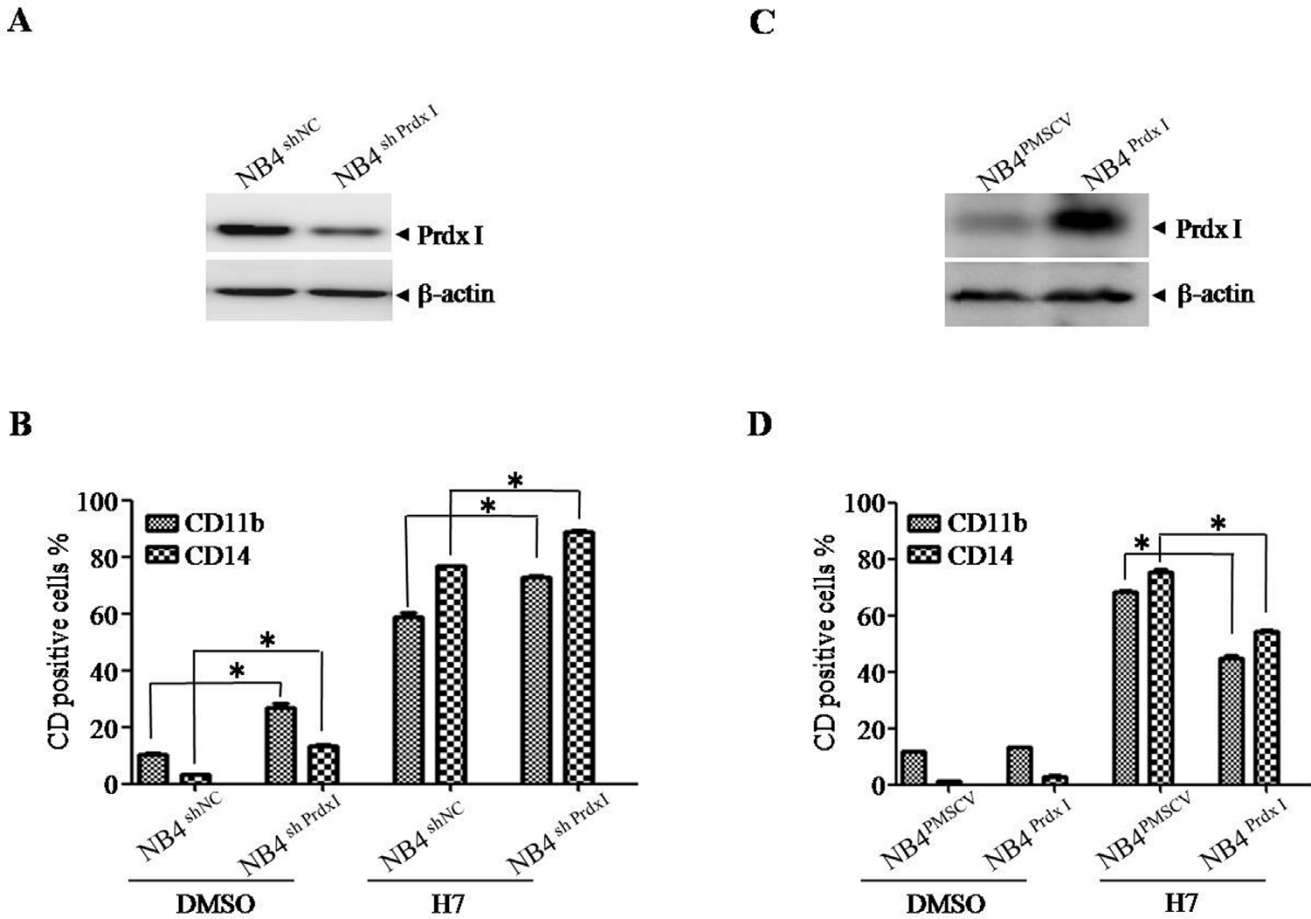
Knockdown or overexpression of Prdx I increases or decreases H7-induced cell differentiation **(A)** The NB4 cells were transfected with non-specific shRNA (NB4^shNC^) or Prdx I specific shRNA (NB4^shPrdx I^). The indicated proteins were examined by Western blot. **(B)** The NB4^shNC^ and NB4^shPrdx I^ cells were treated with H7 for 3 days, and the expression of CD11b, CD14 were determined by FACS. All values represent the means ± S.D. of three independent experiments. *p < 0.05 compared with the control group. **(C)** The NB4 cells were transfected with the vector (NB4^NC^) or Prdx I plasmid (NB4^Prdx I^). The indicated proteins were examined by Western blot. **(D)** The NB4^NC^ and NB4^Prdx I^ cells were treated with H7 for 3 days, and the expression of CD11b and CD14 were determined by FACS. All values represent the means ± S.D. of three independent experiments. *p < 0.05 compared with the control group.

To further confirm the role of Prdx I in H7-induced cell differentiation, Prdx I was knocked down (Figure 4A, NB4^shPrdx I^) or overexpressed in NB4 cells (Figure 4C, NB4^Prdx I^), and nonspecific shRNA- (NB4^shNC^) or control vector-transfected cells (NB4^vector^) were used as negative controls, respectively. Prdx I knockdown induced cell differentiation in NB4 cells, whereas H7 treatment further increased the percentages of CD11b/CD14-positive cells (Figure 4B, Supplementary Figure S2A). By contrast, Prdx I overexpression in NB4 cells partially blocked the differentiation effect of H7 (Figure 4D, Supplementary Figure S2B). These data suggest that Prdx I plays an essential role in H7-induced cell differentiation.

### H7 induces cell differentiation of non-APL leukemia cell lines and primary cells

**Figure 5:**
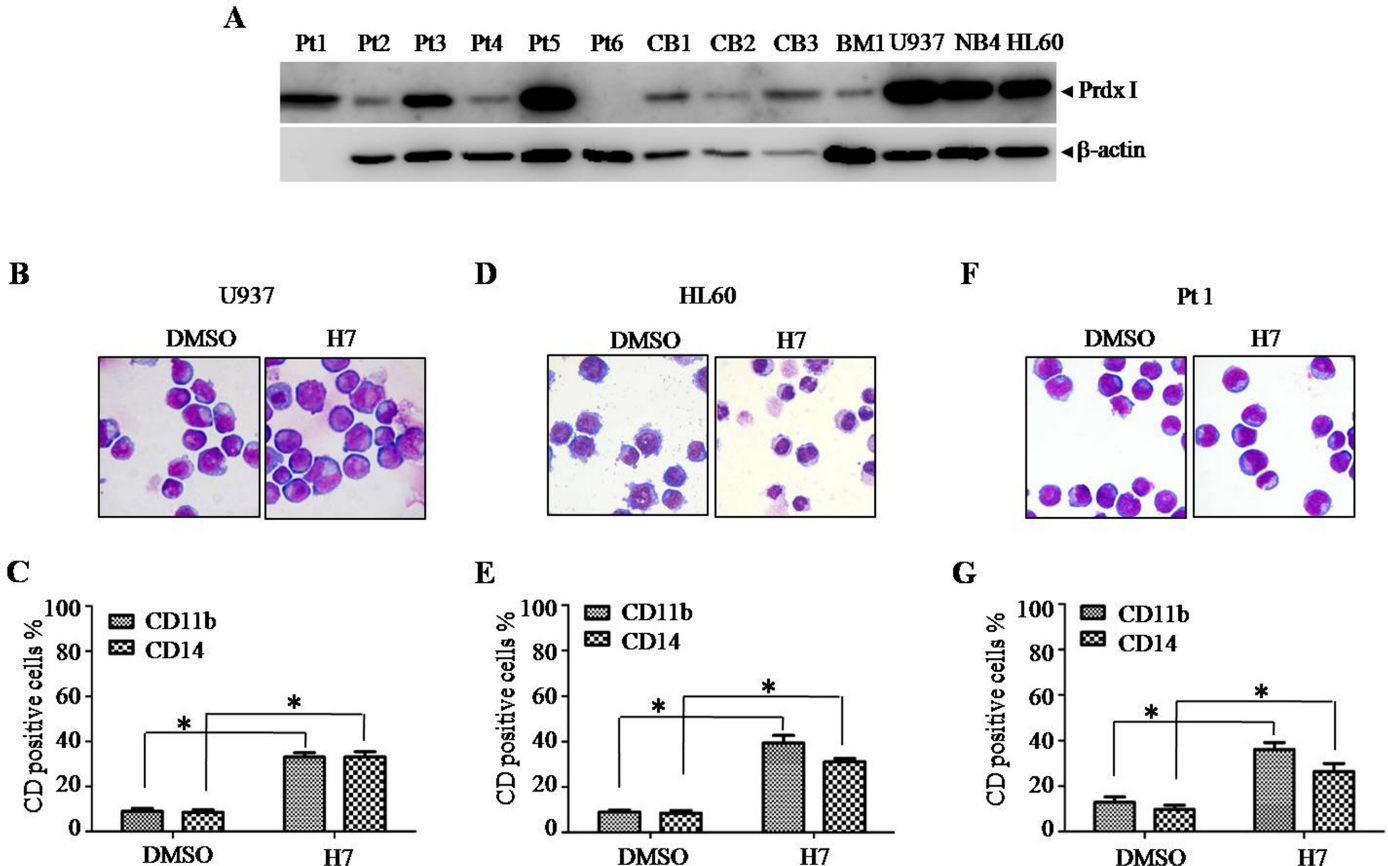
H7 induces monocyte differentiation in U937, HL60, and primary leukemia cells. (A) Prdx I expression in different cell lines and primary cells were determined by Western blot. **(B–F)** The indicated cells were treated with H7 for 3 days, and cell morphology was examined by Wright's staining and light microscopy (100 ×) (B, D, F, day 3). The cell surface markers CD11b and CD14 were detected by FACS, and the percentages of CD11b and CD14 are shown in (C, E, and **G)**. All values represent the means ± S.D. of three independent experiments. *p < 0.05 compared with the control group.

To investigate whether the differentiation-inducing effect of H7 is limited to NB4 cells, we investigated the leukemia cell lines U937 and HL60, the primary leukemia cells, and the normal cord blood or bone marrow mononuclear cells (CBMNCs or BMMNCs). U937, HL60, and NB4 cells expressed high protein level of Prdx I. Varied expression levels of Prdx I were observed in primary leukemia cells. In addition, Prdx I was lowly expressed in normal CBMNCs and BMMNCs (Figure 5A). H7 treatment also induced cell differentiation in U937 (Figure 5B, 5C, Supplementary Figure S3A), in HL60 (Figure 5D, 5E, Supplementary Figure S3B), in some primary leukemia cells (Figure 5F, 5G, Table 1, Supplementary Figure S3C, 3D, 3E), and slightly in CBMNCs and BMMNCs (Table 1, Supplementary Figure S3F, 3G), as shown by the increased CD11b and CD14 expression and/or by the morphological changes in cells. These data suggest that the differentiation-inducing effect of H7 is not limited to APL cells.

**Table 1:** Effect of H7 on differentiation of primary blood cells

	CD11b		CD14	
	DMSO	H7	DMSO	H7
Pt1	12.62 ± 1.45	38.69 ± 1.37*	8.54 ± 0.57	29.57 ± 1.45*
Pt3	8.82 ± 0.20	35.48 ± 0.82*	10.64 ± 0.17	40.78 ± 0.39*
Pt5	7.63 ± 0.16	39.08 ± 0.13*	11.4 ± 3.91	17.23 ± 1.72
CB3	31.56 ± 0.52	43.76 ± 1.87	30.90 ± 0.23	48.15 ± 0.24*
BM1	38.56 ± 0.18	38.90 ± 0.17	23.31 ± 0.56	23.82 ± 0.92

Note: Mononuclear cells from bone marrow or cord blood cells were treated with H7 for 3 days. The percentages of CD11b and CD14 were evaluted by FACS. All values represent means ± S.D. of three independent experiments.

* *p* < 0.05 compared with the DMSO-treated cells.

### H7 induces differentiation through activation of the ROS-Erk1/2-C/EBPβ pathway

**Figure 6:**
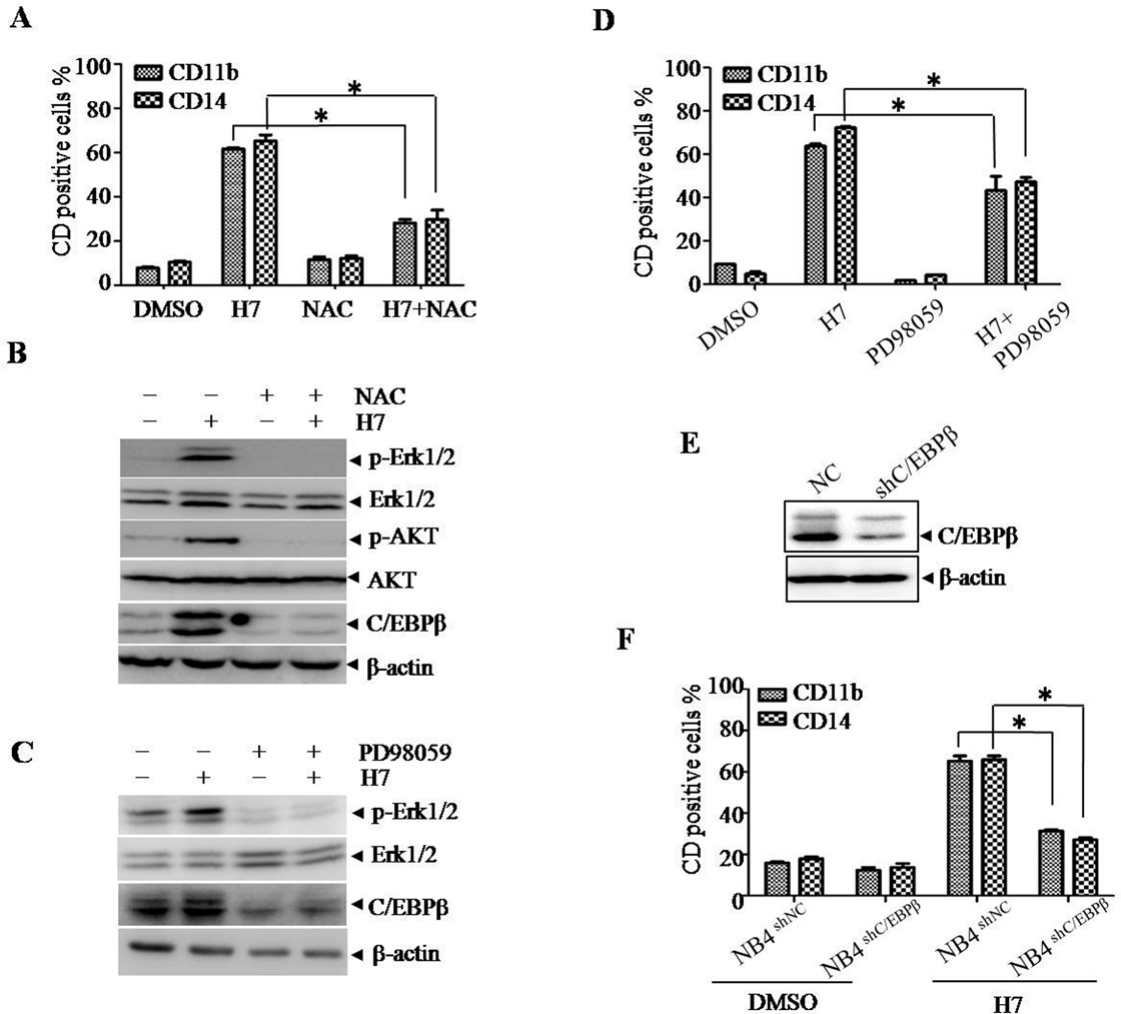
H7 induces monocyte differentiation via ROS-C/EBPβ axis in NB4 cells. (A–B) The NB4 cells were treated with H7 in the presence or absence of NAC for 3 days, and the expression of the cell surface markers CD11b and CD14 was detected by FACS. All values represent the means ± S.D. of three independent experiments. *p < 0.05 compared with the single treatment (A); the indicated proteins were examined by Western blot (B). **(C–D)** The NB4 cells were treated with H7 for 3 days in the presence or absence of PD98059, and the expression levels of the cell surface marker CD11b and CD14 were detected by FACS. All values represent the means ± S.D. of three independent experiments. *p < 0.05 compared with the H7 treatment (D); the indicated proteins were examined by Western blot (C). **(E–F)** The C/EBPβ knockdown NB4 cells (NB4^shC/EBPβ^) and the control NB4 cells (NB4^shNC^) were treated with H7 for 3 days, and the expression of the indicated proteins were detected by Western blot (E); the expression levels of the CD11b^+^ and CD14^+^ were detected by FACS. All values represent the means ± S.D. of three independent experiments. *p < 0.05 compared with the H7-treated NB4^shNC^ cells (F).

Given that the Prdx I inhibitor adenanthin induces leukemia-cell differentiation by increasing ROS level, we hypothesized that H7 induces cell differentiation in a similar manner. To determine the role of ROS in H7-induced cell differentiation, we treated the NB4 cells with H7 in the presence or absence of N-acetyl cysteine (NAC), a ROS scavenger. NAC treatment significantly inhibited H7-induced CD11b and CD14 upregulation (Figure 6A). These data suggest that H7 induces leukemia-cell differentiation in a ROS-dependent manner.

Furthermore, activation of the AKT or MAPK signaling pathway has been implicated in ROS-mediated cell differentiation [23–24]. H7 treatment indeed activated AKT, as evidenced by the increased AKT^Ser473^ phosphorylation (Figure 6B). However, knockdown of AKT expression by shRNA did not affect H7-induced cell differentiation, indicating that AKT activation is not involved in H7-induced cell differentiation (Supplementary Figure S4). Given that Erk1/2 activation is involved in adenanthin-induced differentiation, we then evaluated the possible role of Erk1/2. H7 treatment activated Erk1/2 (Figure 6B). By contrast, co-treatment of H7 with the Erk1/2 inhibitor PD98058 inhibited the H7-induced Erk1/2 activation (Figure 6C) and the CD11b and CD14 upregulation (Figure 6D). These data suggest that Erk1/2 activation is involved in H7-induced cell differentiation.

C/EBPβ is also speculated to drive immature cells to become granulocytes and monocytes and has been shown as an important downstream target of Erk1/2 [25]; thus, we determined the C/EBPβ expression upon H7 treatment. H7 treatment increased C/EBPβ expression, which was abrogated by co-treatment of NAC (Figure 6B). The Erk1/2 inhibitor PD98059 also inhibited the H7-induced C/EBPβ upregulation (Figure 6C). Moreover, knockdown of C/EBPβ (Figure 6E) abrogated the H7-induced CD11b and CD14 upregulation (Figure 6F). These data suggest that H7-induced cell differentiation is mediated through the ROS-Erk1/2-C/EBPβ axis.

### H7 induces leukemia-cell differentiation *in vivo*

**Figure 7:**
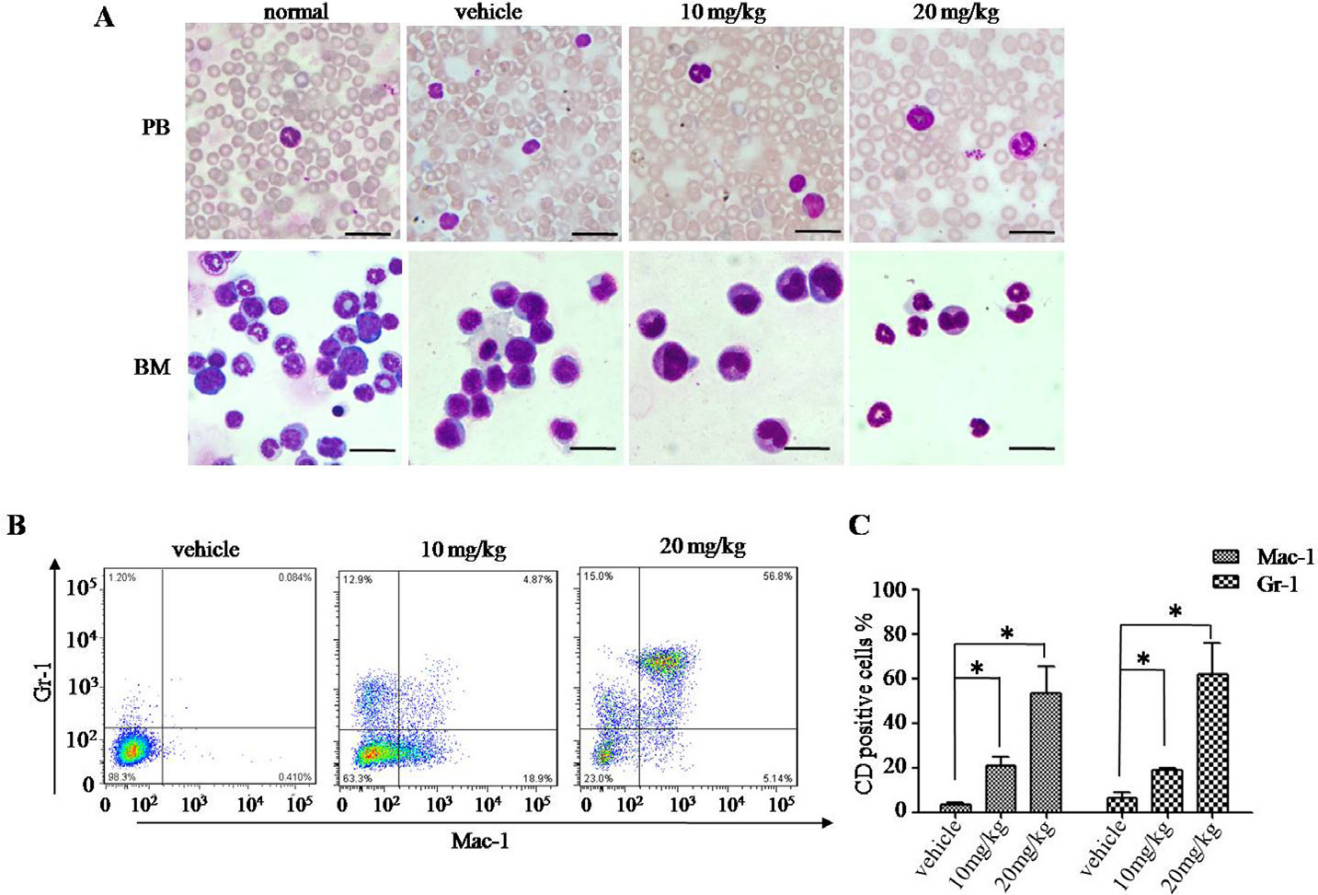
H7 induces leukemia-cell differentiation *in vivo* APL leukemic mice were treated with vehicle and H7 (10 mg/kg, 20 mg/kg, introperitoneal) daily for 5 consecutive days. When the first vehicle-treated leukemic mice were moribund, all mice were killed and analyzed. The cytologic analysis by Wright's staining of the peripheral blood (PB) and bone marrow (BM) are shown in **(A)** Scale bar, 20 μm. The expression levels of Gr-1^+^ and Mac-1^+^ were detected by FACS. The representative images are shown in **(B)** and the percentages of Gr-1^+^ and Mac-1^+^ are shown in **(C)**. All values represent the means ± S.D. of three independent experiments. *p < 0.05 compared with the vehicle-treated mice cells (C).

Given that H7 induces cell differentiation *in vitro*, we then attempted to determine the effect of H7 *in vivo* in our APL mouse model. Compared with the vehicle-treated leukemic mice, the intraperitoneal injection of H7 at 10 and 20 mg/kg increased the terminally differentiated cells in the bone marrow (BM) and peripheral blood (PB), as reflected by the increase in maturating myeloid cells showing differentiation-related morphologic features, such as condensed chromatin with indented, distorted, and horse-shoe- or donut-shaped nuclei (Figure 7A). Moreover, we measured the mouse granulocytic differentiation-related antigens Gr-1 and Mac-1 after gating for myeloid cells by FSC and SSC on flow cytometry. The results showed that in these myeloid cells, the percentages of Gr-1^+^ and Mac-1^+^ cells were much higher in the BM of the H7-treated leukemic mice than those of the vehicle-treated leukemic mice (Figure 7B and 7C). These data indicate that H7 induces leukemia-cell differentiation *in vivo*.

## DISCUSSION

This study found H7 as a novel Prdx I-inhibiting compound and demonstrated that H7 could induce leukemia-cell differentiation. These results have validated that Prdx I is a potential target in inducing leukemia-cell differentiation.

Since the discovery of ROS, the primary focus has been directed to the oxidative damage of biological macromolecules, including proteins, DNA, and lipids. However, accumulating evidence has suggested that changing the cellular ROS level is useful in altering the cell differentiation status [6, 26]. The homeostasis of redox reactions in cells is controlled primarily by antioxidant enzymes, such as catalase, glutathione peroxidase 1, and Prdxs. We recently proposed that targeting the Prdx/ROS axis is a potential novel strategy in inducing leukemia-cell differentiation [20]. However, this hypothesis has not yet been confirmed.

To validate this hypothesis, we first attempted to identify the novel Prdx I inhibitor. We demonstrated that H7 is a novel Prdx I inhibitor, and this finding is supported by several lines of evidence: (A) H7 inhibits Prdx I activity *in vitro*. Moreover, our SPR analysis showed that H7 rapidly associated and disassociated with Prdx I, indicating their non-covalent bonding. The docking study also showed that H7 stably interacted with Prdx I through four hydrogen bonds. (B) H7 binds to Prdx I in cells. Compared with other methods used in confirming the interaction of small molecular compounds with proteins, CETSA can be performed more easily and can directly measure whether a drug molecule reached its targets in cells and animal models [27]. Using this method, we demonstrated that H7 indeed binds to Prdx I in a biologically relevant circumstance. However, H7 could not interact with the four other two-cystein Prdxs (Prdxs II–V), indicating that H7 is relatively selective to Prdx I. The interaction of H7 with Prdx I is further demonstrated by drug affinity responsive target stability (DARTS) assay, which is another established and valid methodology for identifying and studying protein–ligand interactions. Indeed, H7 can protect Prdx I from protease-induced degradation, indicating that H7 may bind to Prdx I (Supplementary Figure S1). (C) Consistent with its Prdx I inhibition activity, H7 treatment increases ROS level in cells.

Using H7 as a chemical probe, we further demonstrated that targeting the Prdx I indeed induced leukemia-cell differentiation. Manipulating the protein level by overexpression or knockdown experiments could decrease or increase H7-induced cell differentiation, further demonstrating that Prdx I is critical in H7-induced cell differentiation. Consistent with our previous report [20], inducing cell differentiation by targeting Prdx I is not limited to NB4 cells. Other non-APL cells and primary leukemia blast cells could also be induced by H7 to differentiate. In addition, H7 treatment could induce cell differentiation in leukemic mice, although H7 failed to prolong the survival of leukemic mice possibly because of its toxicity. Injection of H7 at 20 mg/kg through the tail vein caused mummification necrosis of the tail. By contrast, the mice were more tolerant to intraperitoneal injection of H7, although long-term treatment at 20 mg/kg still resulted in early death of the leukemic mice (data not shown). Decreasing the concentration of H7 to 10 mg/kg will not only reduce its toxicity but also its efficacy. Therefore, optimizing H7 to reduce its toxicity is required. Taken together, these results further confirmed that targeting Prdx I could induce differentiation in APL and non-APL cells.

We previously demonstrated that the Prdx I-ROS axis is vital to the adenanthin-induced cell differentiation [20]. Consistent with this finding, NAC, a blocker of ROS, also inhibited H7-induced leukemia-cell differentiation. This observation suggests that H7 induces leukemia-cell differentiation through the Prdx I-ROS axis. Moreover, the activation of AKT or MAPK signaling pathway has been implicated in ROS-mediated cell differentiation [23–24]. H7 treatment activated AKT and Erk1/2. However, inhibition of Erk1/2 but not of Akt could inhibit H7-induced cell differentiation, indicating that Erk 1/2 activation is involved in the H7-induced cell differentiation. C/EBPβ is also a key transcription factor regulating monocytic gene expression. Several reports and ours have shown that C/EBPβ is an important downstream molecule of Erk1/2 [28–29]. We then investigated the possible role of C/EBPβ in H7-induced leukemia-cell differentiation. As expected, NAC blocked the H7-induced C/EBPβ upregulation in leukemia cells. In addition, silencing of the C/EBPβ protein could weaken the ability of H7 to induce leukemia-cell differentiation. Thus, we proposed that H7 induces leukemia-cell differentiation through the Prdx I-ROS-Erk1/2-C/EBPβ axis.

In conclusion, our findings verified in principle that Prdx I is a novel target for inducing leukemia-cell differentiation. Moreover, H7 is a novel lead compound for optimizing Prdx I inhibition.

## MATERIALS AND METHODS

### Virtual screening

Virtual screening and docking were performed using Glide version 5.5 (Schrodinger Suite 2009) with default docking parameter settings. The Prdx I structure was retrieved from the Protein Data Bank (PDB entry: 1QQ2). Hydrogen atoms and charges were added during a brief relaxation, which was performed using the “Protein Preparation” module in Maestro and executing the “preparation and refinement” option; the restrained partial minimization was terminated when the root-mean-square deviation reached a maximum value of 0.3 Å to relieve the steric clashes of amino acid residues located within 14 Å from the catalytic thiolate of Cys52; these amino acid residues were defined as part of the binding site for docking studies. All crystallographic water molecules were removed from the coordinate set. Moreover, the compound library for screening was obtained from SPECS company [http://www.specs.net/ (October 1, 2013)]. All 180,000 compounds were desalted, neutralized, and parameterized using the OPLS 2005 force field. The tautomers and ionization states expected to occur at 5.0–9.0 pH range were generated using the “ionize” module. During docking, the standard-precision (SP) and extra-precision (XP) dockings were adopted to generate the minimized pose, and the Glide scoring function (G-Score) was used in selecting the final pose exhibiting the lowest energy conformation for each ligand [30]. Thirty-eight compounds from the top 100 compound based on both the SP and XP scores were purchased and dissolved in DMSO for biological testing.

### Prdx I activity assay

Human Prdx I was cloned into pET28a vector containing a His6 tag sequence at the N-terminal region. The proteins were expressed in the *Escherichia coli* strain BL21 and then purified. The Prdx I peroxidase activity was detected by a standard Trx-Trx reductase-NADPH-coupled spectrophotometric assay as described previously [20].

### SPR assay

The SPR experiments were performed on Biacore T200 (GE Healthcare). Full-length human Prdx I was immobilized on a CM5 chip (GE Healthcare) aiming at 500 response units using amine coupling chemistry. Compound binding with Prdx I was analyzed in a single-cycle kinetic analysis at 0.39–25 mM H7 concentration using HBS-P buffer (10 mM HEPES, pH 7.4, 150 mM sodium chloride (NaCl), 0.05% P20) at a flow rate of 30 μl/min at 37°C. Biacore T200 Evaluation Software 1.0 (GE Healthcare) was used in data analysis.

### CETSA

CETSA was performed according to the method described [27]. NB4 cells were harvested and washed with PBS. The cells were diluted in PBS supplemented with complete protease inhibitor cocktail. The cell suspensions were freeze-thawed three times using liquid nitrogen, and the soluble fraction (lysate) was separated from the cell debris by centrifugation at 20,000 g for 20 min at 4°C. The cell lysates were subsequently diluted with PBS and divided into two aliquots; one aliquot was treated with DMSO and the other with H7 diluent. After 10–30 min incubation at room temperature, the respective lysates were divided into smaller (50 μL) aliquots and then heated individually at different temperatures for 3 min (Veriti Thermal Cycler, Applied Biosystems/Life Technologies) followed by cooling for 3 min at room temperature. The appropriate temperatures were determined in preliminary CETSA experiments (data not shown). The heated lysates were centrifuged at 20,000 g for 20 min at 4°C to separate the soluble fractions from the precipitates. The supernatants were transferred into new microtubes and then analyzed by sodium dodecyl sulfate polyacrylamide gel electrophoresis (SDS-PAGE) followed by Western blot analysis.

### DARTS

DARTS is a general methodology for identifying and studying protein–ligand interactions [31] and was performed to investigate the H7–Prdx I interaction. Purified Prdx I protein were incubated with drugs or vehicle at room temperature for 50 min, and the above mixture was digested by pronase (10 mg/ml stock solutions in water-aliquots stored at −20°C) at appropriate ratio dissolved in TNC buffer (50 mM Tris-HCl pH 8.0, 50 mM NaCl, and 10 mM CaCl_2_) for 30 min. The reaction was stopped by adding concentrated SDS-PAGE loading buffer to final 1×, and then mixed well and boiled immediately. The samples were subjected to Western blot.

### Cell culture and agents

The BM samples were collected from newly diagnosed AML patients at the Tongren Hospital and Shanghai First People's Hospital, which are affiliated to Shanghai Jiaotong University School of Medicine (SJTU-SM). Informed consent was obtained from all patients and all manipulations were approved by the Medical Science Ethics Committee of SJTU-SM. The patients were diagnosed according to the French-American-British classification. Patient (Pt) 1 is M3 type, Pt 2–4 and 6 are M1 type, Pt 5 is M5 type. Normal cord blood (CB) or bone marrow (BM) samples were collected from healthy volunteers. In addition, the mononuclear cells were aspirated by Ficoll–Paque liquid. The APL cell line NB4 was obtained from Dr. Michel Lanotte (Hospital Saint Louis, Paris, France), whereas the human monocytic cell line U937 and the human myelomonocytic leukemia cell line HL60 were obtained from the American Type Culture Collection (Manassas, VA). The cells were grown in RPMI-1640 (Bio-Whittaker Europe, Verviers, Belgium) supplemented with 10% fetal calf serum (EuroClone, Life Science Division, Milan, Italy) at 37°C in a humidified atmosphere of 5% CO_2_.

### Cell differentiation assay

The morphological features of the cells were examined by microscopy after Wright's staining (BASO Diagnostic Inc., Guangdong, China) and the cell surface differentiation antigens CD11b and CD14 were measured using fluorescein isothiocyanate- or phycoerythrin-labeled antibodies with isotype controls (Beckman-Coulter, Miami, FL) by flow cytometry.

### Detection of intracellular ROS

The oxidation-sensitive fluorescent probe dye 2′, 7′-dichlorodihydrofluorescein diacetate (DCF-DA, Invitrogen Molecular Probes, Eugene, OR) was used in measuring the intracellular ROS. DCF-DA is deacetylated intracellularly by nonspecific esterases and is further oxidized by cellular peroxides to the fluorescent compound 2′, 7′-dichlorofluorescein. Briefly, the cells treated with or without H7 were washed with phosphate buffered saline (PBS) and then incubated with 20 μM DCF-DA at 37°C for 30 min according to the manufacturer's instructions. The fluorescence signals were detected by a FACStar Flow Cytometer (Beckman Coulter). For each sample, 5,000 or 10,000 events were collected. In addition, the H_2_O_2_ levels were expressed in terms of mean fluorescence intensity.

### RNA interference and transfection

Pairs of complementary oligonucleotides against AKT (5′-GTGGTCATGTACGAGATGA-3′), C/EBPβ [20], and non-target control shRNA were synthesized by Sangon Biotech (Shanghai), annealed, and ligated into the PSIREN-RetroQ Vector (Clontech Laboratories). These shRNA-carrying retroviruses, which were produced in 293T cells, were used to infect the NB4 cells.

### Western blot

Equal volumes of cell lysates were loaded onto 10% SDS–polyacrylamide gel, electrophoresed, and then transferred into ECL-nitrocellulose membranes (Amersham, Buckinghamshire, UK). The membranes were stained with 0.2% Ponceau S red to ensure equal protein loading. After blocking with 5% nonfat milk in Tris-buffered saline, the membranes were incubated with the polyclonal antibodies against Prdxs I–V, C/EBPa, and C/EBPβ (Santa Cruz Biotechnology, Santa Cruz, CA) overnight at 4°C followed by incubation with horseradish peroxidase-linked secondary antibody (cell signaling) for 1 h at room temperature. Detection was performed by SuperSignal West Pico Chemiluminescent Substrate kit (Pierce, Rockford, IL) according to manufacturer's instruction. β-actin (Merck, Darmstadt, Germany) was used as an internal control. Signal intensity of proteins was normalized against β-actin using Quantity One (Bio-Rad).

### Mouse APL model

The leukemia-cells isolated from the spleens of leukemic hMRP8-PML-RARa transgenic mice were intravenously injected into the 6- to 8-week-old female FVB/N mice following sublethal irradiation. Three days after transplantation, the mice were intraperitoneally treated with vehicle (15% Cremophor EL, 15% Propanediol, and 60% PBS) or H7. Cytological and histological analyses were also performed. The Gr-1^+^/Mac-1^+^ cells in BM were determined by FACS. Animal handling was approved by the Committee for Humane Treatment of Animals of SJTU-SM.

### Statistical analysis

Student's *t*-test was used in evaluating the difference between two treatments. A *p* value of less than 0.05 was considered statistically significant.

## SUPPLEMENTARY MATERIAL FIGURES



## Abbreviations

Prdx I: peroxiredoxin I
ATRA: all trans-retinoic acid
ROS: reactive oxygen species
NAC: N-acetyl cysteine
APL: acute promyelocytic leukemia
PBS: phosphate buffered saline
PDB: Protein Data Bank
RMSD: root-mean-square deviation
SP: standard-precision
XP: extra-precision
G-Score: Glide scoring function
CETSA: Cellular Thermal Shift Assay
GPx: glutathione peroxidase 1.
