# Supplementary material for: Identification of H7 as a novel peroxiredoxin I inhibitor to induce differentiation of leukemia cells
Source: Oncotarget. 2015 Dec 26;7(4):3873–83. doi: 10.18632/oncotarget.6763 (PMC4826176; doi:10.18632/oncotarget.6763)
Supplement: Supplementary file 1 [file oncotarget-07-3873-s001.pdf]

Identification of H7 as a novel peroxiredoxin I inhibitor to induce differentiation of leukemia cells

Supplementary Materials

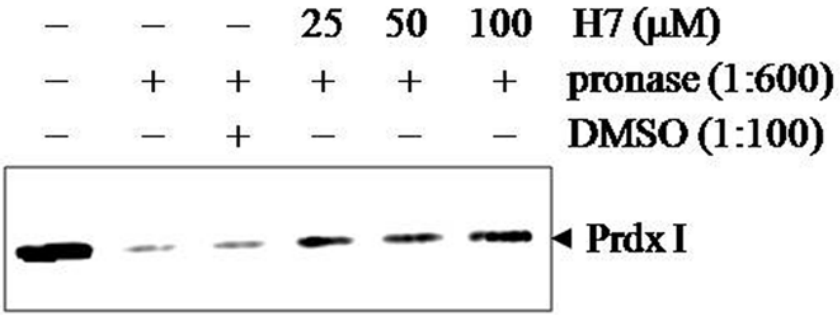

Supplementary Figure S1: H7 interacts with Prdx I. Whole cell lysates of NB4 cells were incubated with H7 followed by digestion with pronase according to DARTS assay described in "Materials and Methods". Then, the degree of Prdx I degradation was determined by western blot. All experiments were repeated for three times.

A

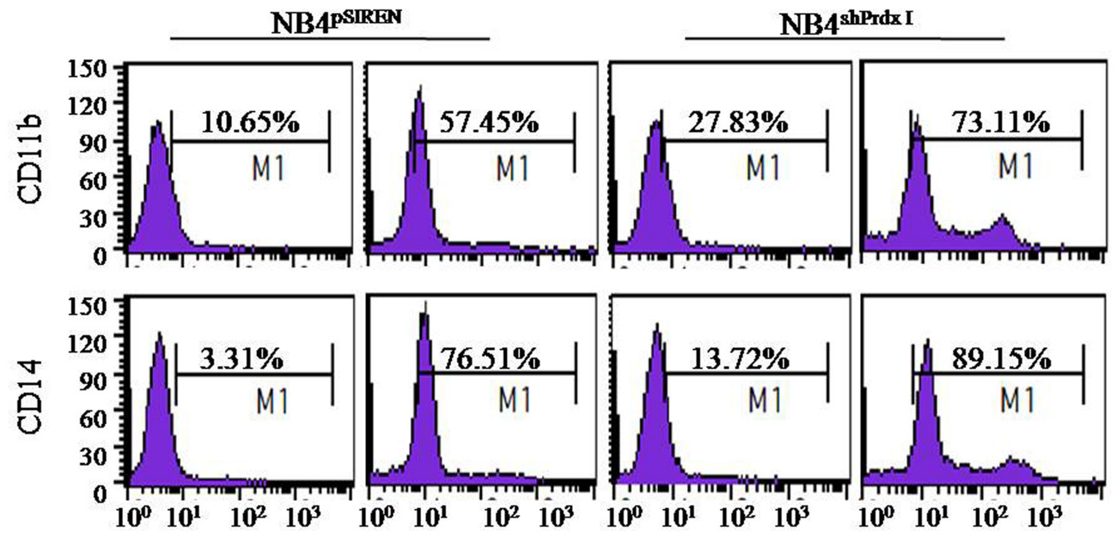

B

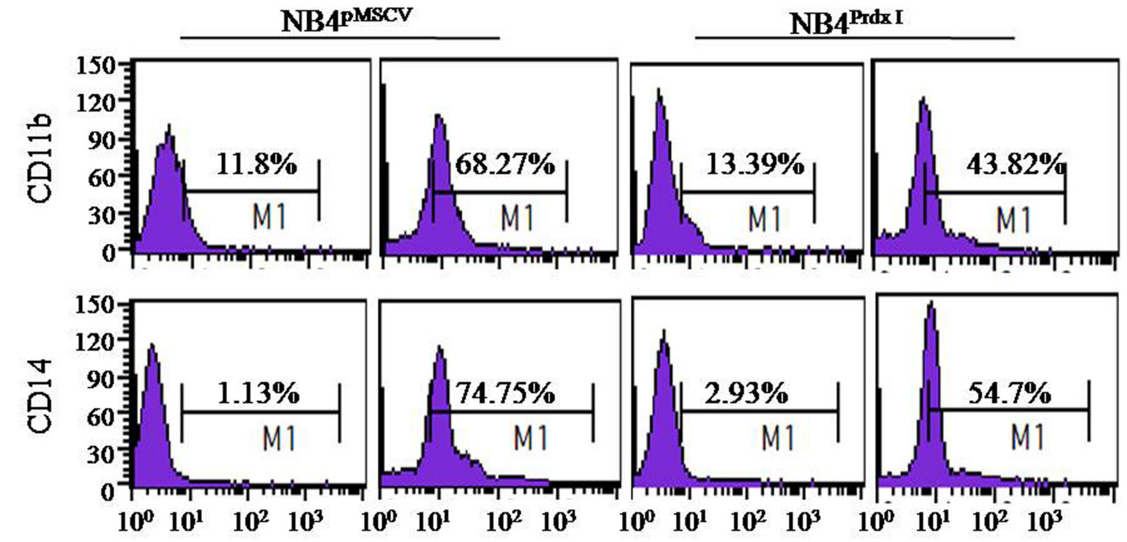

Supplementary Figure S2: Knockdown or overexpression of Prdx I increases or decreases H7-induced cell differentiation. (**A**) The NB4^{shNC} and NB4^{shPrdx} I cells were treated with H7 for 3 days, and the expression of CD11b, CD14 were determined by FACS and the representative histograms were shown. (**B**) The NB4^{NC} and NB4^{Prdx} I cells were treated with H7 for 3 days, and the expression of CD11b and CD14 were determined by FACS. and the representative histograms were shown. M1 The value shown in M1 indicates the percentage of CD11b or CD14 positive cells.

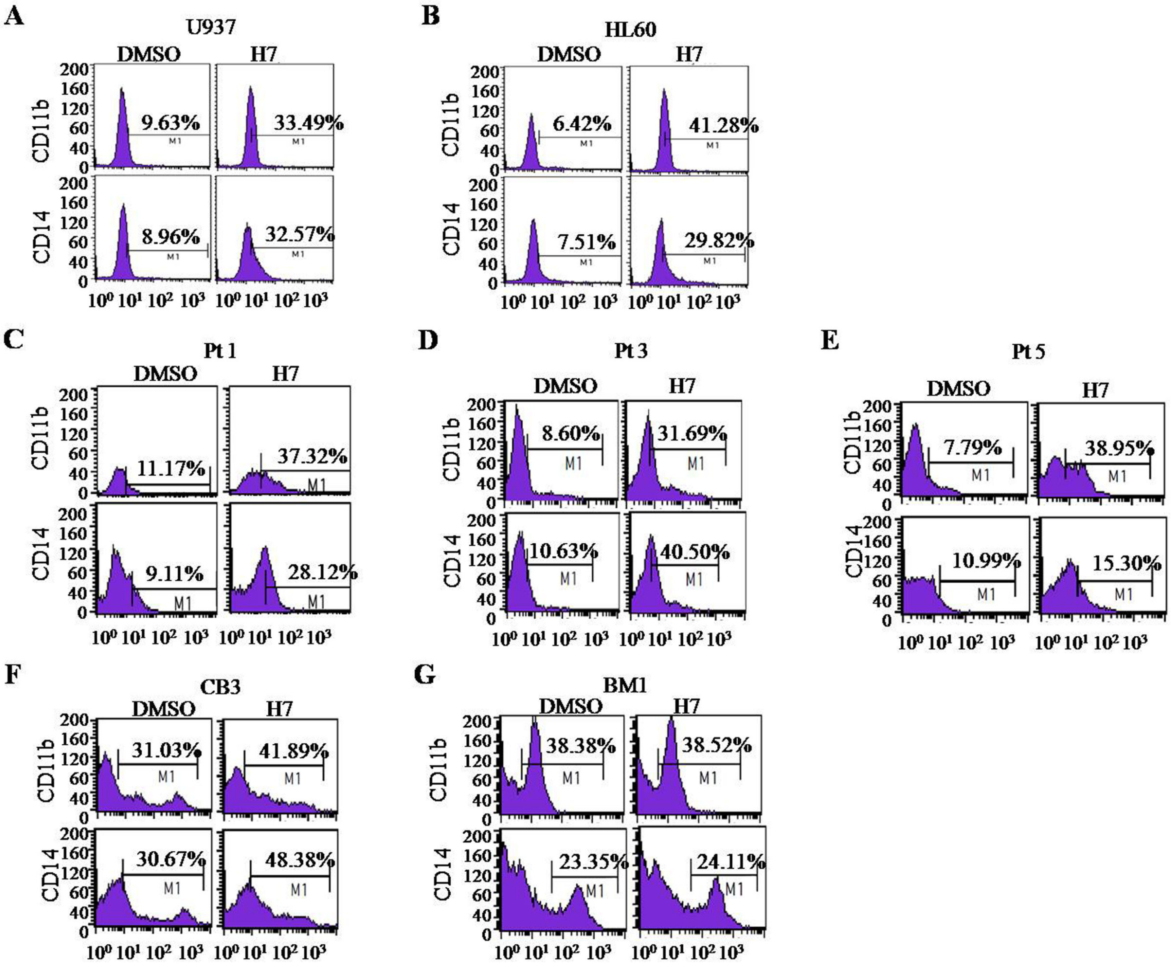

Supplementary Figure S3: H7 induces monocyte differentiation in U937, HL60, and primary leukemia cells. The indicated cells were treated with H7 for 3 days. The expression of CD11b, CD14 were determined by FACS and the representative histograms were shown. The value shown in M1 indicates the percentage of CD11b or CD14 positive cells.

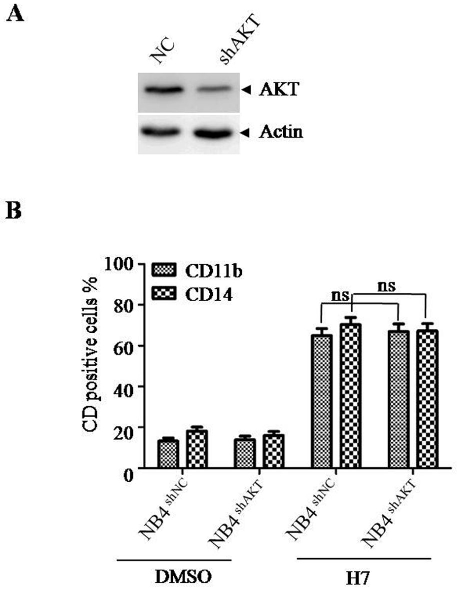

Supplementary Figure S4: Knockdown of AKT has no effect on H7-induced cell differentiation. (**A**) The NB4 cells were transfected with non-specific shRNA ($NB4^{shNC}$) or AKT specific shRNA ($NB4^{shAKT}$). The indicated proteins were examined by Western blot. (**B**) The $NB4^{shNC}$ and $NB4^{shAKT}$ cells were treated with H7 for 3 days, and the expression of CD11b, CD14 were determined by FACS. All values represent the means ± S.D. of three independent experiments. ns, no significant difference.
